# Quality of prostate MRI in early diagnosis—a national survey and reading evaluation

**DOI:** 10.1186/s13244-025-01960-4

**Published:** 2025-04-05

**Authors:** Linda C. P. Thijssen, Jasper J. Twilt, Tristan Barrett, Francesco Giganti, Ivo G. Schoots, Rianne R. M. Engels, Mireille J. M. Broeders, Jelle O. Barentsz, Maarten de Rooij

**Affiliations:** 1 Department of Medical Imaging, Radboud University Medical Center, Nijmegen, The Netherlands; 2 Department of Radiology, Addenbrooke’s Hospital and University of Cambridge, Cambridge, UK; 3 Department of Radiology, University College London Hospital NHS Foundation Trust, London, UK; 4 Division of Surgery and Interventional Science, University College London, London, UK; 5 Department of Radiology & Nuclear Medicine, Erasmus University Medical Centre, Rotterdam, The Netherlands; 6 Department of Radiology, Netherlands Cancer Institute, Amsterdam, The Netherlands; 7 Department for Health Evidence, Radboud University Medical Center, Nijmegen, The Netherlands; 8 Andros Clinics, Arnhem, The Netherlands

**Keywords:** Prostatic neoplasms, Magnetic resonance imaging, Image quality, Netherlands, PI-QUALv2

## Abstract

**Objectives:**

The reliability of image-based recommendations in the prostate cancer pathway is partially dependent on prostate MRI image quality. We evaluated the current compliance with PI-RADSv2.1 technical recommendations and the prostate MRI image quality in the Netherlands. To aid image quality improvement, we identified factors that possibly influence image quality.

**Materials and methods:**

A survey was sent to 68 Dutch medical centres to acquire information on prostate MRI acquisition. The responding medical centres were requested to provide anonymised prostate MRI examinations of biopsy-naive men suspected of prostate cancer. The images were evaluated for quality by three expert prostate radiologists. The compliance with PI-RADSv2.1 technical recommendations and the PI-QUALv2 score was calculated. Relationships between hardware, education of personnel, technical parameters, and/or patient preparation and both compliance and image quality were analysed using Pearson correlation, Mann–Whitney *U*-test, or Student's *t*-test where appropriate.

**Results:**

Forty-four medical centres submitted their compliance with PI-RADSv2.1 technical recommendations, and 26 medical centres completed the full survey. Thirteen hospitals provided 252 usable images. The mean compliance with technical recommendations was 79%. Inadequate PI-QUALv2 scores were given in 30.9% and 50.6% of the mp-MRI and bp-MRI examinations, respectively. Multiple factors with a possible relationship with image quality were identified.

**Conclusion:**

In the Netherlands, the average compliance with PI-RADSv2.1 technical recommendations is high. Prostate MRI image quality was inadequate in 30–50% of the provided examinations. Many factors not covered in the PI-RADSv2.1 technical recommendations can influence image quality. Improvement of prostate MRI image quality is needed.

**Critical relevance statement:**

It is essential to improve the image quality of prostate MRIs, which can be achieved by addressing factors not covered in the PI-RADSv2.1 technical recommendations.

**Key Points:**

- Prostate MRI image quality influences the diagnostic accuracy of image-based decisions.
- Thirty to fifty percent of Dutch prostate MRI examinations were of inadequate image quality.
- We identified multiple factors with possible influence on image quality.

**Graphical Abstract:**

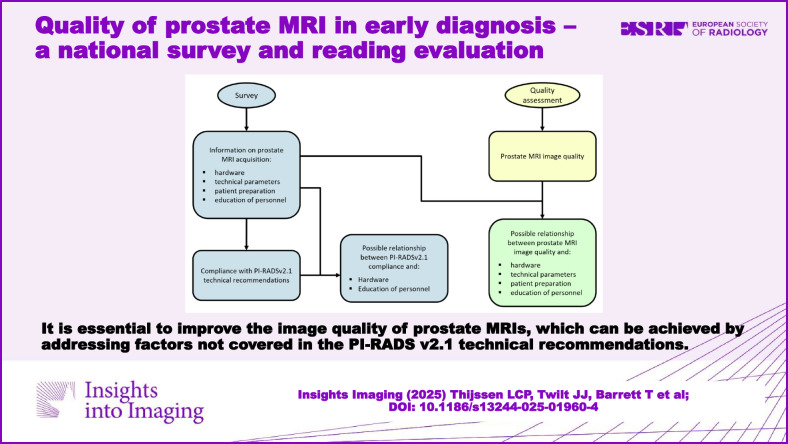

## Introduction

Since 2012, prostate cancer (PCa) has been listed as the second most diagnosed cancer and the fifth cause of cancer-related death in men worldwide [1–3]. With an ageing population, PCa is likely to remain an important health care problem. The PROMIS, PRECISION, 4M, and MR PROPER studies have shown that the MRI pathway for PCa decreases overdiagnosis and overtreatment of indolent PCa, with equal detection of clinically significant PCa while reducing the number of biopsies [4–7]. Due to this benefit, international guidelines were changed and now recommend MRI prior to biopsy [8, 9], which accelerated the uptake of MRI in the PCa pathway [10–12].

However, the variable diagnostic performance in daily practice has raised concerns about the diagnostic accuracy of prostate MRI [13–15]. The performance of MRI for the detection of PCa is strongly dependent on the radiologist’s experience [16, 17] and image quality [13, 17]. Image quality is, in turn, influenced by patient-related factors [16, 18–21], hardware employed [20–22], and technical parameters [20, 21]. The prostate imaging reporting and data system (PI-RADS) version 2.1 recommends minimal technical requirements [23]. The compliance of European and American institutions with these recommendations is highly variable [24–28]. Although several groups have investigated the correlation between PI-RADS technical recommendation compliance and image quality [24, 29], these recommendations are aimed at acquiring acceptable MRI examinations, rather than prescribing technical parameters for optimal image quality.

We used a survey to investigate how prostate MR imaging is performed, and a reader study to determine the current prostate MRI image quality in the Netherlands. Additionally, we evaluated the compliance with PI-RADSv2.1 technical recommendations and explored associations between image quality and patient preparation, education of personnel, MRI hardware, and technical parameters.

## Methods

The institutional review board granted ethical approval and waived written informed consent for the retrospective use of anonymised images.

### Study design

Our observational retrospective study entails three consecutive parts;

Part 1: an online survey to gain insight into the four key domains of prostate MRI acquisition in the Netherlands:

- Hardware;
- Technical parameters;
- Patient preparation;
- Education of personnel.

**Fig. 1:**
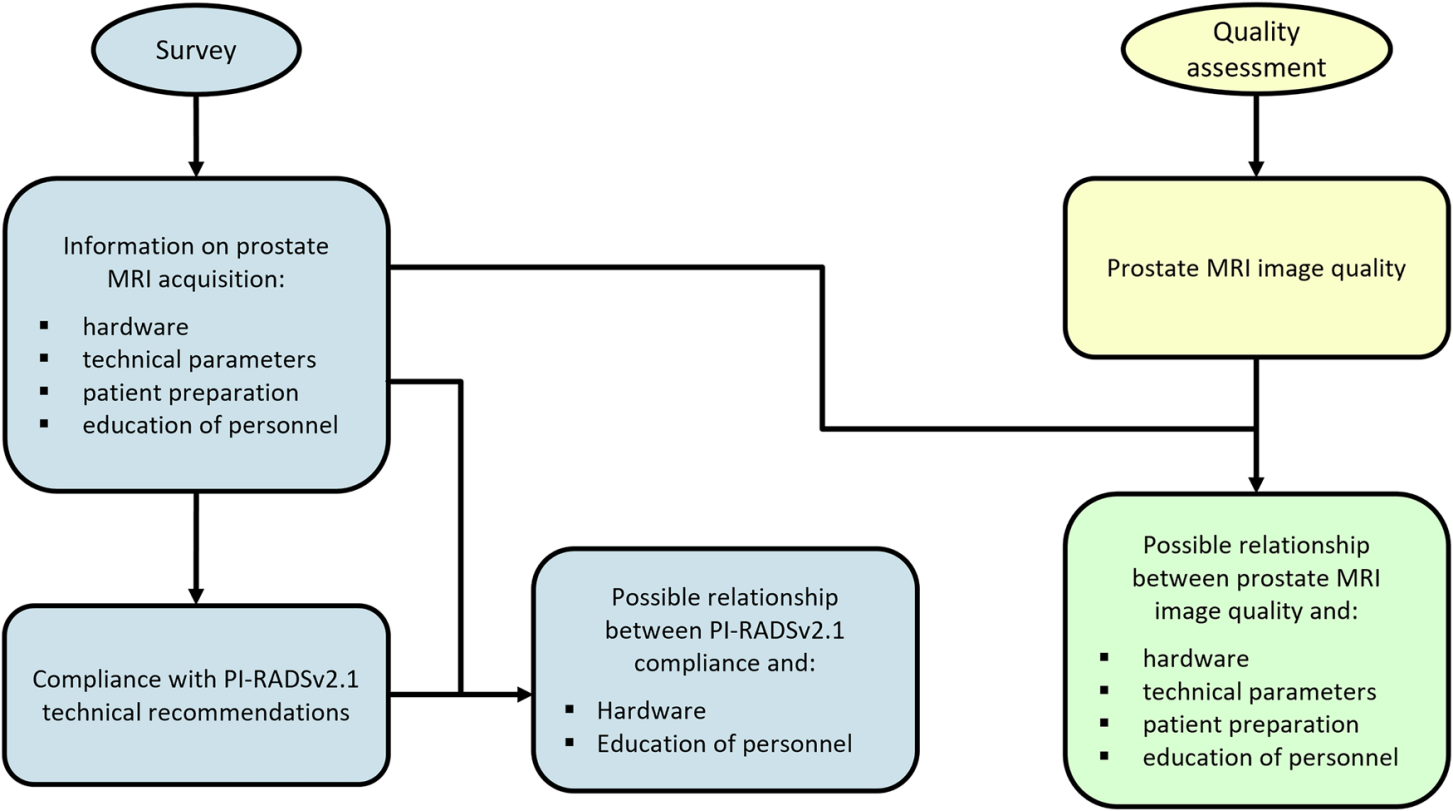
Infographic on study organisation. Blue: part of the study based on a survey. Yellow: part of the study based on the reader study. Green: part of the study based on both survey and reader study

The survey answers were used to assess the compliance with the PI-RADSv2.1 technical recommendations and possible relationships between PI-RADSv2.1 compliance and hardware or education of personnel (Fig. 1, in blue).

Part 2: prostate MRI image quality was assessed in a subset of the responding centres to determine the current state of prostate MRI quality in the Netherlands (Fig. 1, in yellow).

Part 3: The results of both parts were combined to explore associations between image quality and survey answers (Fig. 1, in green).

For the first part, an online survey was developed using LimeSurvey, and tested by a multidisciplinary team from a tertiary prostate MRI centre consisting of prostate radiologists, dedicated radiographers, clinical researchers, and an epidemiologist. The online questionnaire surveyed the above-mentioned four key domains that can influence prostate MRI image quality. See Table 1 and supplementary information for all parameters included in the questionnaire (Supplementary Tables 4–9). In 2020, an invitation containing a unique link to the voluntary online survey was sent by e-mail to the Radiology departments of all hospitals in the Netherlands, excluding a specialist paediatric hospital (*n* = 68). A reminder was sent after two weeks to all hospitals that did not respond or did not fully complete the survey. In case of non-representative or unclear answers, the respondents were contacted by email and asked to verify their answers.

**Table 1:** Compliance per PI-RADS technical recommendation

	PI-RADS v2.1 technical recommendation	compliance, (*n*)
General	T1 with the FOV including pelvic lymph nodes to the aortic bifurcation	57% (25/44)
	Multiparametric acquisition (T2W, DWI, and DCE)	43% (19/44)
	Imaging plane angle, location and slice thickness for all sequences (T2W and DWI for all acquisitions, and additionally DCE only for multiparametric acquisitions) are identical	26% (5/19) (mp), 72% (18/25) (bp), 52% (23/44) (all)
Axial T2W	Additional sagittal and/or coronal	98% (43/44)
	Slice thickness ≤ 3 mm	84% (37/44)
	Interslice gap absent	59% (26/44)
	Field of view 12–20 cm	75% (33/44)
	In-plane resolution (phase) ≤ 0.7 mm	82% (36/44)
	In-plane resolution (frequency) ≤ 0.4 mm	6.8% (3/44)
DWI	Slice thickness ≤ 4 mm	95% (42/44)
	Interslice gap absent	64% (28/44)
	Field of view 16–22 cm	59% (26/44)
	In-plane resolution (phase) ≤ 2.5 mm	82% (36/44)
	In-plane resolution (frequency) ≤ 2.5 mm	82% (36/44)
	Angulation similar to T2	100% (44/44)
	ADC map high *b*-value 800–1000	91% (40/44)
	ADC map low *b*-value 0–100 s/mm^2^	98% (43/44)
	Highest *b*-value ≥ 1400 s/mm^2^	80% (35/44)
DCE	Slice thickness ≤ 3 mm	53% (10/19)
	Interslice gap absent	68% (13/19)
	In-plane resolution (phase) ≤ 2 mm	95% (18/19)
	In-plane resolution (frequency) ≤ 2 mm	95% (18/19)
	Angulation similar to T2	100% (19/19)
	TR < 100 ms	100% (19/19)
	TE < 5 ms	100% (19/19)
	Temporal resolution ≤ 15 s	100% (19/19)
	Total observation time ≥ 2 min	100% (19/19)
	Dose of 0.1 mmol/kg GBCA or equivalent	47% (9/19)
	Injection rate 2–3 mL/s	95% (18/19)

*ADC* apparent diffusion coefficient, *DCE* dynamic contrast-enhanced, *DWI* diffusion weighted imaging, *TE* echo time, *TR* repetition time, *T2W* T2-weighted

For the second part, all hospitals that completed the survey were requested to share the first 20 consecutive prostate MRI examinations acquired that year for image quality assessment. The provided examinations were scored for image quality by 3–5 expert prostate radiologists (> 1000 cases read, > 200 cases/year) [30] from four different international institutions (T.B., I.G.S., J.O.B., F.G., and M.d.R.). The radiologists assessed each available sequence for image quality by using a development version of the Prostate imaging quality v2 scoring system (PI-QUALv2), using quality criteria (Table 2) slightly different from the final version criteria [31].

**Table 2:** Image quality criteria per sequence

T2W	Axial: adequate signal-to-noise ratio in the periprostatic fat
	Axial: ability to clearly delineate relevant prostate structures
	Axial: absence of (severe) artefacts in prostate region
	Sagittal OR coronal: ability to clearly delineate relevant prostate structures
DWI	Adequate signal-to-noise ratio on high *b*-value images (b1400 or higher)
	Adequate range of contrast to identify TZ from PZ on ADC map
	Absence of (severe) artefacts in the prostate region
	DWI in plane matching with T2W imaging (< 5 mm at the posterior prostate)
DCE	Image quality that allows the assessment of enhancement of the prostatic structures
	Ability to identify anatomical structures (for instance, pudendal arteries)

*ADC* apparent diffusion coefficient, *DCE* dynamic contrast-enhanced MRI, *DWI* diffusion weighted imaging, *PZ* peripheral zone, *T2W* T2-weighted, *TZ* transition zone

For the final part, relationships between the quality scores and the survey answers were analysed as described below.

### Statistical analysis

Data was analysed using IBM SPSS Statistics (version 27). For each hospital, the mean quality score for each individual criterion was derived by dividing the sum of the scores assigned by the number of readers who performed the quality assessment for said criterion. Subsequently, the total quality score and total score per sequence were calculated for each centre. Additionally, the PI-QUALv2 score (inadequate/acceptable/optimal) that each reader assigned to each case was calculated. Interrater agreement for image quality assessment was evaluated using the intraclass correlation coefficient (ICC) based on a mean-rating (*k* = 3–5), absolute-agreement, 2-way random-effects model. The reliability of the assessment was considered poor if the ICC was < 0.4, moderate between 0.4 and 0.59, good between 0.6 and 0.75, with an ICC > 0.75 considered excellent. Pearson’s correlation coefficient was used to analyse the correlation between continuous variables. The correlation was considered weak for a Pearson correlation coefficient (*r*) between 0.1 and 0.3, medium between 0.3 and 0.5, and strong between 0.5 and 1.0. Hospitals were divided into groups per feature for analysis of binary variables. To investigate differences in mean image quality and/or compliance between these groups, Mann–Whitney *U*-test was used for small sample sizes where normality assumption could not be met, otherwise, Student’s *t*-test was used. As this extensive analysis was performed to explore possible influencers of image quality and not to prove a causal relationship, no correction for multiple testing was done.

## Results

### Part 1: survey

#### Response

**Fig. 2:**
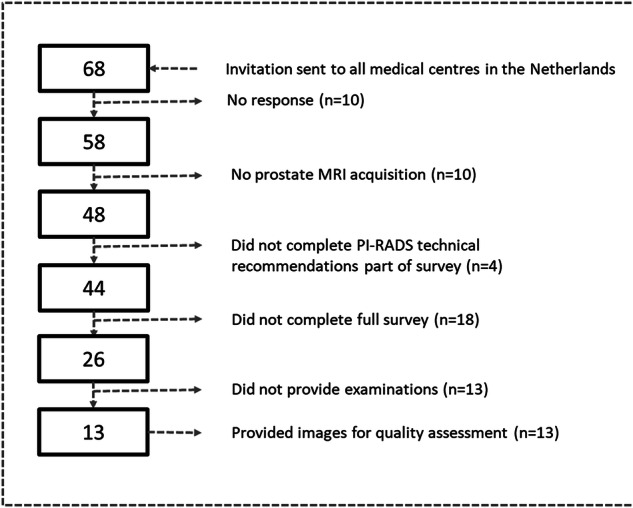
Survey response

Fifty-eight of 68 hospitals (85%) responded, 48 of which (82.8%) performed prostate MRI for the detection of PCa. Forty-four out of 48 (92%) submitted their compliance with PI-RADSv2.1 technical recommendations, and 26/48 (54.2%) centres completed the full survey (Fig. 2 and Tables 1,  3, and 4). Out of these 26 centres, thirteen provided examinations, 6 of which (46%) were multiparametric (mp-)MRI, and 7 (54%) biparametric (bp-)MRI acquisitions. Eight examinations were excluded because of corrupted files or prior prostatectomy, resulting in 140 bp and 112 mp examinations (252 examinations total) for image quality assessment with PI-QUALv2.

**Table 3:** Features showing possible association with image quality according to *t*-test

Feature	Quality aspect	groups	Mean quality (std)		*p*-value	Degrees of freedom	*t*-value
Hardware used							
Field strength	bp quality	1.5 T	5.32/8	(0.56)	0.016	11	−2.85
		3 T	6.23/8	(0.59)			
	T2W quality	1.5 T	2.80/4	(0.39)	0.029	11	−2.52
		3 T	3.30/4	(0.33)			
	DWI quality	1.5 T	2.53/4	(0.32)	0.039	11	−2.34
		3 T	2.93/4	(0.30)			
Ventral coil type^a^	DWI: Adequate range of contrast to identify TZ from PZ on ADC map	Pelvic	0.69/1	(0.15)	0.028	10.88	−2.54
		Body	0.84/1	(0.06)			
Technical settings							
Phase encoding direction sagittal T2W	T2W quality	CC	3.32/4	(0.32)	0.038	10	2.39
		AP	2.83/4	(0.38)			
	T2W sagittal OR coronal: ability to clearly delineate relevant prostate structures	CC	0.87/1	(0.07)	0.019	10	2.80
		AP	0.72/1	(0.11)			
Patient preparation							
Antispasmodic use	T2W axial: adequate signal-to-noise ratio in the periprostatic fat	No	0.53/1	(0.12)	< 0.001	11	−4.74
		Yes	0.82/1	(0.10)			
Refrain from ejaculation	bp quality	No	5.18/8	(0.59)	0.020	11	−2.72
		Yes	6.09/8	(0.58)			
	T2W quality	No	2.72/4	(0.41)	0.033	11	−2.43
		Yes	3.23/4	(0.34)			
	DWI quality	No	2.47/4	(0.31)	0.048	11	−2.23
		Yes	2.86/4	(0.32)			
Use of laxative^b^	T2W axial: adequate signal-to-noise ratio in the periprostatic fat	No	0.72/1	(0.15)	0.038	10	2.39
		Yes	0.35/1	(–)			
	T2W sagittal OR coronal: ability to clearly delineate relevant prostate structures	No	0.79/1	(0.09)	0.012	10	3.08
		Yes	0.52/1	(–)			
Use of rectal catheter^b^	mp quality	No	6.29/10	(0.39)	0.016	3	−4.92
		Yes	8.42/10	(–)			
	bp quality	No	5.52/8	(0.51)	0.014	10	−2.98
		Yes	7.12/8	(–)			
	T2W quality	No	2.91/4	(0.35)	0.031	10	−2.51
		yes	3.82/4	(–)			
	DWI quality	No	2.61/4	(0.28)	0.041	10	−2.35
		Yes	3.29/4	(−)			
	DWI: Adequate signal-to-noise ratio on high *b*-value images (b1400 or higher)	No	0.40/1	(0.13)	0.013	10	−3.00
		Yes	0.80/1	(−)			
Education of personnel							
Education radiologist	bp quality	No	4.47/8	(0.16)	0.031	11	−2.47
		Yes	5.92/8	(0.63)			
	T2W quality	No	2.41/4	(0.11)	0.020	11	−2.72
		Yes	3.14/4	(0.37)			
	T2W axial: adequate signal-to-noise ratio in the periprostatic fat	No	0.42/1	(0.09)	0.006	11	−3.37
		Yes	0.76/1	(0.14)			

Only correlations with a *p*-value < 0.05 are included in this table. See supplementary information for a full table

*ADC* apparent diffusion coefficient, *AP* anterior-posterior, *bp* biparametric, *CC* craniocaudal, *DWI* diffusion weighted imaging, *mp* multiparametric, *PZ* peripheral zone, *T2W* T2-weighted imaging, *TZ* transition zone

^a^ Levene’s test for equality of variances was found to be violated for this analysis (*F*(1, 10) = 0.6.41, *p* = 0.028). Owing to this violated assumption, a *t*-statistic not assuming homogeneity of variance was computed

^b^ Feature is used by a single hospital

**Table 4:** Features showing Pearson correlation with image quality

Feature	Quality aspect	Degrees of freedom	Correlation coefficient (*r*)	*p*-value
Technical settings				
Interslice gap T2W axial^a^	T2W axial: adequate signal-to-noise ratio in the periprostatic fat	11	−0.57	0.040
Resolution T2W axial—frequency direction	Total T2W quality	11	−0.64	0.019
	T2W axial: ability to clearly delineate relevant prostate structures	11	−0.69	0.009
Slice thickness T2W sagittal	T2W sagittal OR coronal: ability to clearly delineate relevant prostate structures	11	−0.58	0.038
Field of view T2W sagittal phase direction	T2W sagittal OR coronal: ability to clearly delineate relevant prostate structures	11	0.65	0.016
Interslice gap DWI	Total DWI quality	11	0.67	0.013
	DWI: adequate SNR on high *b*-value images	11	0.78	0.002
Highest *b*-value	DWI in plane matching with T2W	11	0.80	0.001

Only correlations with a *p*-value < 0.5 are included in this table. See Supplementary Information for a full table *T2W* T2-weighted imaging

^a^ The negative correlation is caused by an extreme outlier

#### PI-RADSv2.1 technical recommendations

The median compliance with PI-RADSv2.1 technical recommendations for the hospitals that completed the survey was 74% (interquartile range (IQR) 70–79%), similar to the compliance of the subset of hospitals that provided prostate MRI examinations for image quality assessment (median 78%, (IQR 72–83%). The median compliance per hospital for mp-MRI was 79% (IQR 76–86%) and 72% (IQR 67–78%) for bp-MRI.

The T2W recommendations had a median compliance of 78% (IQR 46–88%), with lower compliance for the absence of an interslice gap, and in particular, for resolution in the frequency direction. The average compliance for DWI recommendations was 86% (IQR 68–97%), with a high compliance for slice thickness and apparent diffusion coefficient (ADC) map values and low compliance for the absence of an interslice gap and field of view. The mean compliance for dynamic contrast enhanced (DCE) recommendations was 95% (IQR 68–100%), with low compliance for absence of an interslice gap, slice thickness, and gadolinium-based contrast agent dose. The general PI-RADSv2.1 recommendations had a mean compliance of 52%, with notably low compliance for identical plane angle, location and slice thickness for mp-MRI (Table 2).

### Part 2: image quality assessment

#### Interrater agreement and image quality

Interrater agreement for image quality assessment was moderate to good (ICC 0.55–0.62). The mean image quality was 3.0 ± 0.9/4 (mean ± std/number of criteria) for T2 weighted imaging (T2W) acquisitions, 2.8 ± 0.8/4 (mean ± std/number of criteria) for diffusion weighted imaging (DWI) acquisitions, and 1.1 ± 0.5/2 (mean ± std/number of criteria) for DCE acquisition. The mean total quality was 7.3 ± 1.5/10 (mean ± std/number of criteria) for mp-MRI acquisitions, and 5.8 ± 1.5/8 (mean ± std/number of criteria) for bp-MRI acquisitions. For the mp-MRI cases, an optimal PI-QUALv2 score was given in 20.2%, 48.9% scored acceptable, and 30.9% inadequate. The bp-cases scored optimal in 15.6%, acceptable in 33.8% and inadequate in 50.6%. Although PI-QUALv2 states mp-MRI quality must be evaluated with DCE images [31], we did score the mp-cases as bp-cases to determine to what extent the lower percentage of inadequate cases for mp-MRI was due to the DCE sequences providing a ‘safety net’. When the mp-cases were scored as bp-cases, 30.0% of the cases scored optimal, 29.5% acceptable, and 40.5% scored inadequate for PI-QUALv2 image quality.

### Part 3: survey results in four key domains and possible associations with image quality

#### Hardware

Mp-MRI was performed by 43% of the hospitals (19/44, Table 1). The most common field strength was 3 Tesla (T) (60%, 29/48), while 40% of the respondents (19/48) used 1.5 T. The mean age of the scanners was 5.3 years (range 0–11). None of the centres used an endorectal coil.

Possible associations were found between several image quality aspects and hardware. A 3 T field strength had higher image quality scores than 1.5 T for total bp-MRI quality, total T2W quality, and total DWI quality. The body coil had a higher score for “DWI: Adequate range of contrast to identify TZ from PZ on ADC map” than the pelvic coil. We found a possible medium Pearson correlation between scanner age and general recommendations compliance (*r*(40) = −0.315, *p* = 0.042) and DWI recommendations (*r*(40) = −0.340, *p* = 0.028), but no indication for an association between compliance and field strength.

#### Technical parameters

When the phase encoding direction for the sagittal T2W scan was craniocaudal instead of anterior-posterior, higher quality scores were given for “T2W sagittal or coronal: ability to clearly delineate relevant prostate structures” and total T2W quality (Table 3). The size of the T2W axial interslice gap had a strong negative correlation with the quality score for adequate SNR, while the resolution for these images had a strong negative correlation with the ability to delineate relevant structures and the total T2 quality. For sagittal T2W images, the slice thickness was strongly negatively correlated with the ability to delineate relevant prostate structures, while the FOV in phase direction was strongly positively correlated with this ability. The DWI interslice gap showed a strong positive correlation with the adequate SNR on high *b*-value images, and total DWI quality. The value of the highest *b*-value was strongly positively correlated with the quality score for DWI in-plane matching with T2W (Table 4).

#### Patient preparation

The patient preparation methods most commonly employed were antispasmodic agents (64.6% always, 31/48; 10.4% on indication, 5/48), and refraining from ejaculation (38.3% always, 18/48). Other patient preparation methods (i.e. laxatives, enemas, rectal catheters and dietary prescriptions) were only used sporadically (< 5%) (Table 3).

The use of an antispasmodic agent, refraining from ejaculation prior to the scan, and the use of a rectal catheter seem to positively influence several image quality aspects. The use of a laxative possibly has a negative influence on two T2W image quality aspects (Table 3).

#### Education of personnel

The majority of radiologists in the studied centres followed a dedicated educational course on prostate MRI (80.9%, 38/47). In 13 of these 38 centres, both radiologists and radiographers completed a course on prostate MRI (27.7%, 13/47).

There was no correlation between the compliance with PI-RADSv2.1 technical recommendations and the attendance of a prostate MRI course by a radiographer and/or radiologist. Hospitals with radiologists who participated in a course dedicated to prostate MRI scored higher on T2W SNR, total T2W quality, and total bp quality (Table 3).

## Discussion

We assessed how prostate MRI is performed in the Netherlands by conducting an online survey in Dutch hospitals and evaluated the image quality of prostate MRI images in a subset of the respondents. We analysed the collected data to identify factors that potentially influence image quality.

### Compliance with PI-RADSv2.1 technical recommendations

We found a high, but variable adherence to PI-RADSv2.1 technical recommendations by parameter and by hospital, similar to the adherence found by previous groups [24, 26, 27], but higher than that reported by Coskun et al [25]. Notably, there was low adherence of 6.8% to the recommendation “T2W in-plane resolution (frequency) ≤ 0.4 mm”. This may be due to the inverse relationship between resolution and SNR. Therefore, it may be challenging to adhere to this recommendation without loss of image quality or significantly increasing scan time to compensate for quality loss, particularly at 1.5 T. Another explanation could be that centres did not update their protocols after the 2015 publication of PI-RADSv2 [32], as a significantly higher percentage 84% (37/44) was adherent to the PI-RADSv1 [33] recommendation of an in-plane resolution of 0.5–0.7 mm in frequency direction.

### Image quality

The overall image quality in this study, according to PI-QUALv2, was inadequate in 50.6% of bp-MRI and 30.9% of mp-MRI examinations. The number of studies of inadequate quality is relatively high, and if prostate MRI is to remain the preferred first step in PCa detection in the Netherlands, the overall MRI image quality must improve. Several studies have shown that when modifying the scanner protocol according to centre-specific expert recommendations, significant improvements in image quality can be achieved [34, 35]. Although PI-RADSv2 recommends mp-MRI, the necessity of the DCE sequence has been debated, at least for certain patient populations, such as in a screening setting. If the radiologist is experienced in reading prostate MRI [36], and the T2W and DWI images are of adequate image quality [37, 38], DCE does not seem to offer an advantage that justifies the increased costs, scan time or risks of adverse reactions [39]. In our sample, the average T2W and DWI image quality of the hospitals that perform bp-MRI is lower than that of the hospitals that perform mp-MRI. It raises concerns that the hospitals that would potentially benefit the most from DCE imaging refrain from acquiring that sequence.

### Hardware

The positive influence of higher field strength on image quality is explained by the higher SNR [40] and has been reported previously [41]. The possible influence of surface coil type on the range of contrast seems counterintuitive. The body coil demonstrated a higher score for this quality aspect compared to a pelvic coil, despite the pelvic coil being marketed as being superior for prostate imaging. This may be explained by differences in the number of channels employed, which we did not quantify in the study.

### Technical parameters

The negative effect of an interslice gap on image quality for axial T2W images was unexpected, since an interslice gap reduces slice cross-talk. Further analysis of the data revealed this correlation was due to a single extreme outlier; the centre with the lowest quality score reported an interslice gap of 15 mm. When this centre was excluded from the analysis, there was a very weak positive Pearson correlation between interslice gap and the axial T2W SNR (*r*(10) = 0.161, *p* = 0.617).

Some technical parameters that are mentioned in the PI-RADSv2.1 technical recommendations (i.e. absence of interslice gap and small FOV) seem to have a negative influence on quality when the parameters are (nearly) compliant with the recommended value. This shows that the PI-RADSv2.1 technical recommendations are meant to achieve adequate coverage and detailed images of the prostate, not to optimise image quality.

### Patient preparation

The use of an antispasmodic agent to reduce image degradation due to bowel movement had a positive influence on T2W adequate SNR ratio in the periprostatic fat, consistent with prior studies [42, 43]. The use of a rectal catheter to evacuate gas from the rectum seemed to have a positive effect on DWI SNR. This reduction of air diminishes susceptibility artefacts and may also reduce peristalsis and thereby motion artefacts. However, it is important to note that a rectal catheter was used by only one hospital, and this correlation could be based on chance.

### Education of personnel

In the hospitals where the radiologist followed a dedicated course on reporting prostate MRI, the images received a higher quality score. In the past, prostate imaging courses tended to focus mainly on prostate MRI interpretation, whereas the acquisition and image quality of prostate MRIs were covered to a lesser extent. After the introduction of a standardised scoring system for prostate image quality assessment, there has been an increasing awareness of prostate image quality. Both a UK consensus meeting [44] and an expert panel [45] recommend prostate MRI course participation in certification of prostate MRI radiologists. Prostate MRI educational courses should not solely focus on interpretation, but cover quality assessment as well, and include knowledge of potential causative factors to enable radiologist to adapt their protocols to obtain improved image quality. Recent studies have shown that courses on assessing prostate MRI quality improve the application of the PI-QUALv1 scoring system [46–48].

### Limitations

Our study has some important limitations. As with any online survey, we relied on the accuracy and reliability of the respondents’ answers. Furthermore, it is possible that only the hospitals that were confident in their image quality chose to submit their images, leading to an overestimation of image quality. Although our survey covered multiple facets, image quality may have been influenced by additional features such as gradient strength, slew rate, number of activated coils, and the use of denoising filters. These unexplored variables could provide valuable insights in future research.

Despite these limitations, our study provides important insights into the current state of prostate MRI image quality and highlights areas for potential improvement in imaging protocols and radiologist training.

## Conclusion

In the Netherlands, there is a good availability of hospitals that perform prostate MRI. On average, these hospitals have high compliance with PI-RADSv2.1 technical recommendations. However, 30–50% of the studies of the 13 centres that submitted images were considered of inadequate image quality. Adherence to the PI-RADSv2.1 technical recommendations alone does not automatically result in acceptable or optimal image quality, as quality is influenced by other factors such as hardware, patient preparation, and technical settings, which are not specifically addressed in the PI-RADSv2.1 technical recommendations. Awareness of prostate image quality and continued education of personnel are key factors in delivering an adequate image-based diagnostic pathway of PCa.

## Supplementary information

ELECTRONIC SUPPLEMENTARY MATERIAL

## Data Availability

The authors do not have permission to share the data obtained from other medical centres.

## Abbreviations

ADC: Apparent diffusion coefficient
bp: Biparametric
DCE: Dynamic contrast enhanced
DWI: Diffusion weighted imaging
ICC: Intraclass correlation coefficient
mp: Multiparametric
PCa: Prostate cancer
PI-QUAL: Prostate imaging quality scoring system
PI-RADS: Prostate imaging reporting and data system
T: Tesla
T2W: T2 weighted imaging
