# Supplementary material for: Quality of prostate MRI in early diagnosis—a national survey and reading evaluation
Source: Insights Imaging. 2025 Apr 5;16:82. doi: 10.1186/s13244-025-01960-4 (PMC11972232; doi:10.1186/s13244-025-01960-4)
Supplement: Supplementary file 1 — ELECTRONIC SUPPLEMENTARY MATERIAL [file 13244_2025_1960_MOESM1_ESM.pdf]

Quality of prostate MRI in early diagnosis – a national survey and reading evaluation

ELECTRONIC SUPPLEMENTARY MATERIAL

Table S1: T-test analysing associations with PI-RADS technical recommendation compliance

Feature	Compliance subgroup	Groups	Mean compliance (std)	p-value	Degrees of freedom	t-value
Radiologist followed a course on prostate MRI	General, T2W, DWI and DCE (mp-hospitals only)	No	.69 (-)	0.18	16	-1.41
		Yes	.81 (.08)			
Radiographer followed a course on prostate MRI in addition to radiologist	DCE recommendations	No	.88 (.09)	0.88	15	-1.59
		Yes	.89 (.11)			
	General, T2W, DWI and DCE (mp-hospitals only)	No	.82 (.10)	0.51	15	.67
		Yes	.79 (.06)			
Both radiographer and radiologist followed a course on prostate MRI	DCE recommendations	No	.88 (.09)	0.92	16	-.10
		Yes	.89 (.11)			
	General, T2W, DWI and DCE (mp-hospitals only)	No	.81 (.10)	0.74	16	.34
		Yes	.79 (.06)			
Field strength	General, T2W, DWI and DCE (mp-hospitals only)	1.5T	.78 (.10)	0.44	17	-.80
		3T	.81 (.07)			

Table S2: Mann-Whitney U-test analysing associations with PI-RADS technical recommendation compliance

Feature	Compliance subgroup	groups	Mean rank	Exact sig (2 tailed)	U
Radiologist followed a course on prostate MRI	General recommendations	No	17.44	0.21	112
		Yes	23.21		
	T2W recommendations	No	20.56	0.74	140
		Yes	22.38		
	DWI recommendations	No	23.00	0.79	144
		Yes	21.74		
	DCE recommendations	No	11.00	1.00	7
		Yes	9.41		
	General. T2W and DWI (bp-hospitals only)	No	13.50	0.84	64
		Yes	12.76		
Radiographer followed a course on prostate MRI in addition to radiologist	general recommendations	No	19.75	0.054	82.5
		Yes	13.38		
	T2W recommendations	No	15.41	0.08	86
		Yes	21.33		
	DWI recommendations	No	18.18	0.58	117
		Yes	16.25		
	General. T2W and DWI (bp-hospitals only)	No	8.50	0.53	19.5
		Yes	10.63		
Both radiographer and radiologist followed a course on prostate MRI	general recommendations	No	23.55	0.16	138
		Yes	18.00		
	T2W recommendations	No	19.95	0.07	122.5
		Yes	27.29		
	DWI recommendations	No	22.68	0.56	165
		Yes	20.25		
	General. T2W and DWI (bp-hospitals only)	No	12.57	0.55	33
		Yes	15.25		
Field strength	general recommendations	1.5T	23.67	0.61	213
		3T	21.69		
	T2W recommendations	1.5T	18.50	0.06	162
		3T	25.27		
	DWI recommendations	1.5T	22.58	0.97	232.5
		3T	22.44		
	DCE recommendations	1.5T	9.08	0.67	33.5
		3T	10.42		
	General. T2W and DWI (bp-hospitals only)	1.5T	12.21	0.61	68.5
		3T	13.73		

Table S3: Pearson correlation with PI-RADS technical recommendation compliance

Feature	Compliance subgroup	p- value	Correlation coefficient (r)	Degrees of freedom
Scanner age	General recommendations	0.04	-.315	40
	T2W recommendations	0.84	-.033	40
	DWI recommendations	0.03	-.340	40
	DCE recommendations	0.32	-.257	15
	General. T2W and DWI (bp-hospitals only)	0.07	-.365	23
	General, T2W, DWI and DCE (mp-hospitals only)	0.30	-.267	15

Table S4: Pearson correlation between mean image quality and hardware + technical settings

Feature	Quality aspect	Degrees of freedom	Correlation coefficient (r)	p-value
Hardware				
MRI scanner age	Total mp quality	4	.20	0.70
	Total bp quality	11	.19	0.54
	Total T2W quality	11	.23	0.46
	T2W axial: adequate SNR	11	.20	0.51
	T2W axial: ability to delineate	11	.35	0.25
	T2W axial: absence of artefacts	11	.14	0.66
	T2W sag/cor: ability to delineate	11	-.00	0.99
	Total DWI quality	11	.10	0.74
	DWI: adequate SNR high b-value	11	.05	0.87
	DWI: adequate range of contrast	11	.11	0.73
	DWI: absence of artefacts	11	.07	0.83
	DWI in plane matching with T2W	11	.05	0.88
	Total DCE quality	4	-.34	0.51
	DCE: assessment of enhancement	4	-.42	0.41
	DCE: ability to identify	4	-.24	0.65
Technical settings				
Slice thickness T2W axial	Total T2W quality	11	0.22	0.48
	T2W axial: adequate SNR	11	0.02	0.95
	T2W axial: ability to delineate	11	0.32	0.29
	T2W axial: absence of artefacts	11	0.20	0.52

Slice thickness T2W sagittal	Total T2W quality	11	-0.40	0.18
	T2W sag/cor: ability to delineate	11	-.58	0.04
Slice thickness T2W coronal	Total T2W quality	10	0.15	0.65
	T2W sag/cor: ability to delineate	10	0.11	0.73
Interslice gap T2W axial	Total T2W quality	11	-0.41	0.161
	T2W axial: adequate SNR	11	-.57	0.040
	T2W axial: ability to delineate	11	-0.19	0.532
	T2W axial: absence of artefacts	11	0.24	0.422
Interslice gap T2W Sagittal	Total T2W quality	11	0.18	0.553
	T2W sag/cor: ability to delineate	11	0.01	0.966
Interslice gap T2W coronal	Total T2W quality	10	-0.03	0.918
	T2W sag/cor: ability to delineate	10	-0.15	0.647
Field of view T2W axial read direction	Total T2W quality	11	-0.33	0.276
	T2W axial: adequate SNR	11	-0.24	0.424
	T2W axial: ability to delineate	11	-0.05	0.863
	T2W axial: absence of artefacts	11	-0.46	0.115
Field of view T2W sagittal read direction	Total T2W quality	11	0.34	0.250
	T2W sag/cor: ability to delineate	11	0.55	0.050
Field of view T2W coronal read direction	Total T2W quality	10	0.24	0.447
	T2W sag/cor: ability to delineate	10	0.43	0.168
Field of view T2W axial phase direction	Total T2W quality	11	-0.33	0.276
	T2W axial: adequate SNR	11	-0.24	0.424
	T2W axial: ability to delineate	11	-0.05	0.863
	T2W axial: absence of artefacts	11	-0.46	0.115
Field of view T2W sagittal phase direction	Total T2W quality	11	0.42	0.149
	T2W sag/cor: ability to delineate	11	.65	0.016
Field of view T2W coronal phase direction	Total T2W quality	10	0.48	0.111
	T2W sag/cor: ability to delineate	10	0.45	0.138
Resolution T2W axial phase direction	Total T2W quality	11	-0.41	0.163
	T2W axial: adequate SNR	11	-0.14	0.645
	T2W axial: ability to delineate	11	-0.52	0.069
	T2W axial: absence of artefacts	11	-0.36	0.232
Resolution T2W sagittal phase direction	Total T2W quality	11	0.13	0.677
	T2W sag/cor: ability to delineate	11	0.04	0.898
Resolution T2W coronal phase direction	Total T2W quality	10	0.19	0.562
	T2W sag/cor: ability to delineate	10	0.02	0.958
	Total T2W quality	11	-.64	0.019

Resolution T2W axial – frequency direction	T2W axial: adequate SNR	11	-0.33	0.276
	T2W axial: ability to delineate	11	-.69	0.009
	T2W axial: absence of artefacts	11	-0.445	0.128
Resolution T2W sagittal – frequency direction	Total T2W quality	11	0.177	0.562
	T2W sag/cor: ability to delineate	11	0.156	0.611
Resolution T2W coronal – frequency direction	Total T2W quality	10	0.223	0.486
	T2W sag/cor: ability to delineate	10	0.162	0.616
Slice thickness DWI	Total DWI quality	11	-0.157	0.609
	DWI: adequate SNR high b-value	11	-0.493	0.087
	DWI: adequate range of contrast	11	-0.228	0.455
	DWI: absence of artefacts	11	0.403	0.172
	DWI in plane matching with T2W	11	0.280	0.355
Interslice gap DWI	Total DWI quality	11	.667	0.013
	DWI: adequate SNR high b-value	11	.783	0.002
	DWI: adequate range of contrast	11	0.232	0.446
	DWI: absence of artefacts	11	0.205	0.502
	DWI in plane matching with T2W	11	0.414	0.160
Field of view DWI – read direction	Total DWI quality	11	0.170	0.578
	DWI: adequate SNR high b-value	11	0.217	0.475
	DWI: adequate range of contrast	11	0.179	0.558
	DWI: absence of artefacts	11	0.126	0.682
	DWI in plane matching with T2W	11	-0.210	0.490
Field of view DWI – Phase direction	Total DWI quality	11	0.367	0.218
	DWI: adequate SNR high b-value	11	0.387	0.192
	DWI: adequate range of contrast	11	0.033	0.914
	DWI: absence of artefacts	11	0.372	0.210
	DWI in plane matching with T2W	11	0.158	0.606
Resolution DWI – Phase direction	Total DWI quality	11	-0.097	0.751
	DWI: adequate SNR high b-value	11	0.325	0.278
	DWI: adequate range of contrast	11	-0.258	0.395
	DWI: absence of artefacts	11	-0.471	0.105
	DWI in plane matching with T2W	11	-0.081	0.792
Resolution DWI –	Total DWI quality	11	-0.126	0.681

Frequency direction	DWI: adequate SNR high b-value	11	0.321	0.285
	DWI: adequate range of contrast	11	-0.303	0.314
	DWI: absence of artefacts	11	-0.491	0.089
	DWI in plane matching with T2W	11	-0.090	0.770
Lowest b-value	Total DWI quality	11	0.046	0.882
	DWI: adequate SNR high b-value	11	0.089	0.774
	DWI: adequate range of contrast	11	0.018	0.953
	DWI: absence of artefacts	11	0.180	0.555
	DWI in plane matching with T2W	11	-0.245	0.419
Highest b-value	Total DWI quality	11	0.401	0.174
	DWI: adequate SNR high b-value	11	0.233	0.443
	DWI: adequate range of contrast	11	-0.124	0.687
	DWI: absence of artefacts	11	0.428	0.144
	DWI in plane matching with T2W	11	.797	0.001
Number of b-values	Total DWI quality	11	0.024	0.937
	DWI: adequate SNR high b-value	11	0.177	0.563
	DWI: adequate range of contrast	11	-0.373	0.209
	DWI: absence of artefacts	11	0.047	0.878
	DWI in plane matching with T2W	11	0.274	0.365
DCE Slice thickness	Total DCE quality	4	-0.320	0.537
	DCE: assessment of enhancement	4	-0.499	0.314
	DCE: ability to identify	4	-0.136	0.797
DCE interslice gap	Total DCE quality	4	-0.003	0.995
	DCE: assessment of enhancement	4	0.029	0.956
	DCE: ability to identify	4	-0.032	0.952
DCE Resolution – Phase direction	Total DCE quality	4	0.082	0.877
	DCE: assessment of enhancement	4	0.036	0.946
	DCE: ability to identify	4	0.117	0.825
DCE resolution – Frequency direction	Total DCE quality	4	0.252	0.630
	DCE: assessment of enhancement	4	0.208	0.693
	DCE: ability to identify	4	0.272	0.602
DCE Repetition time	Total DCE quality	4	-0.148	0.779
	DCE: assessment of enhancement	4	-0.329	0.524
	DCE: ability to identify	4	0.024	0.964
DCE excitation time	Total DCE quality	4	-0.704	0.119

	DCE: assessment of enhancement	4	-0.705	0.118
	DCE: ability to identify	4	-0.649	0.163
DCE temporal resolution	Total DCE quality	4	0.403	0.429
	DCE: assessment of enhancement	4	0.635	0.176
	DCE: ability to identify	4	0.165	0.755
DCE: field of view (read direction)	Total DCE quality	4	-.44	.381
	DCE: assessment of enhancement	4	-.37	.469
	DCE: ability to identify	4	-.47	.347
DCE: field of view (phase direction)	Total DCE quality	4	-.08	.877
	DCE: assessment of enhancement	4	-.25	.632
	DCE: ability to identify	4	.07	.890

Table S5: T-test analysing association between mean image quality and hardware

Feature	Quality aspect	t-value	Degrees of freedom	p-value	groups	Total quality	std
Hardware used							
Manufacturer	Total mp quality	.32	4	.766	Siemens	7.12	1.18
					Phillips	6.81	1.21
	Total bp quality	-.13	11	.897	Siemens	5.72	.76
					Phillips	5.78	.74
	Total T2W quality	.47	11	.646	Siemens	3.08	.48
					Phillips	2.96	.40
	T2W axial: adequate SNR	-.61	11	.552	Siemens	.68	.21
					Phillips	.75	.14
	T2W axial: ability to delineate	1.59	11	.141	Siemens	.74	.14
					Phillips	.62	.13
	T2W axial: absence of artefacts	.52	11	.614	Siemens	.85	.10
					Phillips	.82	.10
	T2W sag/cor: ability to delineate	Due to a violated normality assumption. The Mann-Whitney test was performed (Table 5)					
	Total DWI quality	-.86	11	.411	Siemens	2.64	.35
					Phillips	2.82	.40
	DWI: adequate SNR high b-value	-2.04	11	.066	Siemens	.38	.19
					Phillips	.58	.12
	DWI: adequate range of contrast	.13	11	.902	Siemens	.74	.12
					Phillips	.73	.20
	DWI: absence of artefacts	.77	11	.455	Siemens	.75	.12
					Phillips	.70	.09
	DWI in plane matching with T2W	Due to a violated normality assumption. Mann-Whitney test was performed (Table 5)					
	Total DCE quality	-.14	4	.899	Siemens	1.04	.34
					Phillips	1.08	.40
					Siemens	.57	.13

	DCE: assessment of enhancement	-.09	4	.931	Phillips	.59	.22
	DCE: ability to identify	-.16	4	.879	Siemens	.46	.21
					Phillips	.49	.19
Field Strength	Total mp quality	-1.89	4	.132	1.5T	6.28/10	.47
					3T	7.64/10	1.15
	Total bp quality	-2.85	11	.016	1.5T	5.32/8	.56
					3T	6.23/8	.59
	Total T2W quality	-2.52	11	.029	1.5T	2.80/4	.39
					3T	3.30/4	.33
	T2W axial: adequate SNR	-1.66	11	.125	1.5T	.63/1	.19
					3T	.79/1	.13
	T2W axial: ability to delineate	-1.87	11	.089	1.5T	.63/1	.14
					3T	.77/1	.12
	T2W axial: absence of artefacts	-2.01	11	.070	1.5T	.80/1	.10
					3T	.90/1	.07
	T2W sag/cor: ability to delineate	-1.91	11	.083	1.5T	.73/1	.12
					3T	.85/1	.09
	Total DWI quality	-2.34	11	.039	1.5T	2.53/4	.32
					3T	2.93/4	.30
	DWI: adequate SNR high b-value	-1.61	11	.136	1.5T	.38/1	.13
					3T	.54/1	.22
	DWI: adequate range of contrast	-1.46	11	.173	1.5T	.68/1	.16
					3T	.80/1	.11
	DWI: absence of artefacts	-1.16	11	.270	1.5T	.70/1	.13
					3T	.77/1	.08
	DWI in plane matching with T2W	Due to a violated normality assumption. Mann-Whitney test was performed (Table 5)					
	Total DCE quality	-.45	4	.675	1.5T	.99/2	.28
					3T	1.12/2	.42
	DCE: assessment of enhancement	-.54	4	.618	1.5T	.54/1	.10
					3T	.62/1	.23
	DCE: ability to identify	-.34	4	.752	1.5T	.45/1	.19
					3T	.51/1	.21
Ventral coil type[1]	Total bp quality	.63	11	.540	pelvic	5.83	.79
					Body/torso	5.55	.61

	Total T2W quality	.70	11	.498	pelvic	3.09	.47
					Body/torso	2.90	.38
	T2W axial: adequate SNR	1.35	11	.205	pelvic	.75	.19
					Body/torso	.61	.10
	T2W axial: ability to delineate	.11	11	.912	pelvic	.70	.15
					Body/torso	.69	.13
	T2W axial: absence of artefacts	.72	11	.486	pelvic	.86	.10
					Body/torso	.81	.11
	T2W sag/cor: ability to delineate	-.10	11	.919	pelvic	.78	.15
					Body/torso	.79	.06
	Total DWI quality	.42	11	.682	pelvic	2.74	.38
					Body/torso	2.65	.35
	DWI: adequate SNR high b-value	1.04	11	.319	pelvic	.49	.20
					Body/torso	.38	.15
	DWI: adequate range of contrast[2]	-2.54	10.88	.028	pelvic	.69	.15
					Body/torso	.84	.06
	DWI: absence of artefacts	.38	11	.714	pelvic	.74	.10
					Body/torso	.72	.14
	DWI in plane matching with T2W	2.02	11	.068	pelvic	.82	.06
					Body/torso	.72	.12

Since none of the hospitals used an endorectal coil the influence of this coil on image quality could not be analyzed.

[1] All mp-hospitals use body type ventral coils. Therefore, no influence of this feature on DCE quality could be investigated.

[2] Levene’s test for equality of variances was violated for this analysis F(1.11)=6.76. p=.025. Due to this violated assumption. a t statistic not assuming homogeneity of variances was computed.

Table S6: T-test analysing association between mean image quality and technical settings

Feature	Quality aspect	t-value	Degrees of freedom	p-value	groups	Total quality	std
Technical settings							
Phase encoding direction T2W axial	Total T2W quality	.09	11	.931	AP	3.07/4	-
					RL	3.03/4	.45
	T2W axial: adequate SNR	.76	11	.466	AP	.84/1	-
					RL	.69/1	.18
	T2W axial: ability to delineate	-.05	11	.962	AP	.69/1	-
					RL	.70/1	.15
	T2W axial: absence of artefacts	-1.01	11	.336	AP	.75/1	-
					RL	.85/1	.10
Phase encoding direction T2W sagittal	Total T2W quality	2.39	10	.038	HF	3.32/4	.32
					AP	2.83/4	.38
	T2W sag/cor: ability to delineate	2.80	10	.019	HF	.87/1	.07
					AP	.72/1	.11
Phase encoding direction T2W coronal	Total T2W quality	-.20	9	.850	HF	3.07/4	-
					RL	3.15/4	.39
	T2W sag/cor: ability to delineate	-.38	9	.713	HF	.79/1	-
					RL	.83/1	.08
Measure method high b-value	Total DWI quality	.01	11	.996	Scanned	2.71/4	.44
					Calculated	2.71/4	.31
	DWI: adequate SNR high b-value	1.41	11	.185	Scanned	.53/1	.15
					Calculated	.39/1	.20
	DWI: adequate range of contrast	.05	11	.961	Scanned	.74/1	.18
					Calculated	.73/1	.13
	DWI: absence of artefacts	-2.01	11	.069	Scanned	.68/1	.11
					Calculated	.78/1	.09
	DWI in plane matching with T2W	-.70	11	.497	Scanned	.77/1	.13
					Calculated	.80/1	.05
DWI: Phase encoding direction	Total DWI quality	-.84	11	.418	AP	2.60/4	.35
					RL	2.78/4	.38
					AP	.37/1	.15

	DWI: adequate SNR high b-value	-1.43	11	.181	RL	.51/1	.20
	DWI: adequate range of contrast	.33	11	.750	AP	.75/1	.13
					RL	.72/1	.17
	DWI: absence of artefacts	-.16	11	.878	AP	.73/1	.14
					RL	.74/1	.10
	DWI in plane matching with T2W	-.89	11	.392	AP	.76/1	.13
					RL	.80/1	.06
DCE: Phase encoding direction	Total DCE quality	-.11	4	.915	AP	1.03/2	.51
					RL	1.07/2	.31
	DCE: assessment of enhancement	.39	4	.715	AP	.62/1	.27
					RL	.56/1	.14
	DCE: ability to identify	-.57	4	.600	AP	.41/1	.24
					RL	.51/1	.18

Table S7: T-test analysing association between mean image quality and patient preparation methods

Feature	Quality aspect	t-value	Degrees of freedom	p-value	groups	Total quality	std
Patient preparation							
Administration of antispasmodic[1]	Total bp quality	-1.29	11	.225	Never	5.42/8	.62
					Always. …	5.94/8	.75
	Total T2W quality	-2.00	11	.071	Never	2.76/4	.37
					Always. …	3.20/4	.40
	T2W axial: adequate SNR	-4.74	11	<.001	Never	.53/1	.12
					Always. …	.82/1	.10
	T2W axial: ability to delineate	-.67	11	.515	Never	.66/1	.12
					Always. …	.72/1	.16
	T2W axial: absence of artefacts	-.45	11	.662	Never	.83/1	.10
					Always. …	.85/1	.11
	T2W sag/cor: ability to delineate	-1.00	11	.340	Never	.74/1	.14
					Always. …	.81/1	.11
	Total DWI quality	-.34	11	.741	Never	2.67/4	.33
					Always. …	2.74/4	.40
	DWI: adequate SNR high b-value	-1.29	11	.222	Never	.37/1	.15
					Always. …	.51/1	.20
	DWI: adequate range of contrast[2]	1.30	10.93	.220	Never	.79/1	.09
					Always. …	.70/1	.17
	DWI: absence of artefacts	.23	11	.824	Never	.74/1	.13
					Always. …	.73/1	.10
	DWI in plane matching with T2W	-.88	11	.397	Never	.76/1	.13
					Always. …	.80/1	.06
Ask patient to refrain from ejaculation days prior to scan	Total mp quality[3]	-1.18	3.77	.306	No	6.37/10	.64
					Yes	7.26/10	1.21
	Total bp quality	-2.72	11	.020	No	5.18/8	.59
					Yes	6.09/8	.58
	Total T2W quality	-2.43<br>-2.33	11	.033<br>.051	No	2.72/4	.41
					Yes	3.23/4	.34
	T2W axial: adequate SNR	-1.35	11	.204	No	.62/1	.21

					Yes	.76/1	.15
	T2W axial: ability to delineate	-2.87	11	.015	No	.58/1	.13
					Yes	.77/1	.10
	T2W axial: absence of artefacts	-1.26	11	.233	No	.80/1	.11
					Yes	.87/1	.09
	T2W sag/cor: ability to delineate	-1.89	11	.085	No	.71/1	.13
					Yes	.83/1	.09
	Total DWI quality	-2.23	11	.048	No	2.47/4	.31
					Yes	2.86/4	.32
	DWI: adequate SNR high b-value	-1.34	11	.207	No	.37/1	.14
					Yes	.51/1	.20
	DWI: adequate range of contrast	-1.24	11	.243	No	.67/1	.18
					Yes	.77/1	.12
	DWI: absence of artefacts	-2.04	11	.067	No	.67/1	.12
					Yes	.78/1	.08
	DWI in plane matching with T2W	-.86	11	.407	No	.76/1	.14
					Yes	.80/1	.05
	Total DCE quality[4]	.66	3.37	.555	No	1.15/2	.08
					Yes	1.01/2	.41
	DCE: assessment of enhancement[5]	.24	3.08	.825	No	.60/1	.02
					Yes	.57/1	.21
	DCE: ability to identify[6]	.91	3.99	.415	No	.55/1	.09
					Yes	.44/1	.22
Administration of laxative	Total bp quality	1.22	10	.252	Never	5.72/8	.66
					Always. …	4.88/8	-
	Total T2W quality	1.75	10	.110	Never	3.05/4	.39
					Always. …	2.33/4	-
	T2W axial: adequate SNR	2.39	10	.038	Never	.72/1	.15
					Always. …	.35/1	-
	T2W axial: ability to delineate	.86	10	.408	Never	.70/1	.15
					Always. …	.57/1	-
	T2W axial: absence of artefacts	-.68	10	.514	Never	.83/1	.10
					Always. …	.90/1	-
	T2W sag/cor: ability to delineate	3.08	10	.012	Never	.79/1	.09
					Always. …	.52/1	-

	Total DWI quality	.34	10	.739	Never	2.67/4	.35
					Always. …	2.55/4	-
	DWI: adequate SNR high b-value	1.78	10	.105	Never	.45/1	.15
					Always. …	.17/1	-
	DWI: adequate range of contrast	.55	10	.596	Never	.74/1	.16
					Always. …	.65/1	-
	DWI: absence of artefacts	-1.20	10	.258	Never	.72/1	.11
					Always. …	.85/1	-
Use of a rectal catheter	Total mp quality	-4.92	3	.016	Never	6.29/10	.39
					Always. …	8.42/10	-
	Total bp quality	-2.98	10	.014	Never	5.52	.51
					Always. …	7.12	-
	Total T2W quality	-2.51	10	.031	Never	2.91	.35
					Always. …	3.82	-
	T2W axial: adequate SNR	-1.77	10	.107	Never	.66	.16
					Always. …	.96	-
	T2W axial: ability to delineate	-2.06	10	.067	Never	.67	.13
					Always. …	.95	-
	T2W axial: absence of artefacts	-1.45	10	.178	Never	.82	.09
					Always. …	.96	-
	T2W sag/cor: ability to delineate	-1.73	10	.115	Never	.76	.11
					Always. …	.95	-
	Total DWI quality	-2.35	10	.041	Never	2.61	.28
					Always. …	3.29	-
	DWI: adequate SNR high b-value	-3.00	10	.013	Never	.40	.13
					Always. …	.80	-
	DWI: adequate range of contrast	-.65	10	.532	Never	.72	.16
					Always. …	.83	-
	DWI: absence of artefacts	-.84	10	.421	Never	.72	.11
					Always. …	.82	-
	DWI in plane matching with T2W	-.86	10	.408	Never	.77	.09
					Always. …	.85	-
	Total DCE quality	-1.35	3	.269	Never	.90/2	.29
					Always. …	1.34/2	-
	DCE: assessment of enhancement	-1.38	3	.261	Never	.50/1	.12

					Always. …	.68/1	-
	DCE: ability to identify	-1.28	3	.292	Never	.40/1	.18
					Always. …	.66/1	-
Use the toilet to empty the bladder immediately prior to scan	Total mp quality	.27	4	.799	No	7.15/10	1.46
					Yes	6.87/10	1.10
	Total bp quality	-.37	11	.716	No	5.68/8	.69
					Yes	5.84/8	.84
	Total T2W quality	-.66	11	.521	No	2.96/4	.42
					Yes	3.13/4	.49
	T2W axial: adequate SNR	-1.94	11	.078	No	.64/1	.18
					Yes	.82/1	.12
	T2W axial: ability to delineate	-.05	11	.959	No	.69/1	.11
					Yes	.70/1	.20
	T2W axial: absence of artefacts	.17	11	.868	No	.85/1	.10
					Yes	.84/1	.11
	T2W sag/cor: ability to delineate	.08	11	.936	No	.79/1	.13
					Yes	.78/1	.12
	Total DWI quality	.04	11	.969	No	2.71/4	.35
					Yes	2.71/4	.42
	DWI: adequate SNR high b-value	-1.34	11	.207	No	.40/1	.19
					Yes	.54/1	.16
	DWI: adequate range of contrast[7]	1.11	5.47	.313	No	.78/1	.10
					Yes	.67/1	.19
	DWI: absence of artefacts	1.02	11	.329	No	.76/1	.12
					Yes	.70/1	.09
	DWI in plane matching with T2W	-.37	11	.722	No	.78/1	.11
					Yes	.80/1	.05
	Total DCE quality	-.11	4	.915	No	1.03/2	.51
					Yes	1.07/2	.31
	DCE: assessment of enhancement	.39	4	.715	No	.62/1	.27
					Yes	.56/1	.14
	DCE: ability to identify	-.57	4	.600	No	.41/1	.24
					Yes	.51/1	.18

[1] All mp-hospitals use an antispasmodic. Therefore, no influence of this feature on DCE quality could be investigated. [2,3,4,5,6,7] Levene's test for equality of variances was violated for this analysis. Due to this violated assumption. a t statistic not assuming homogeneity of variances was computed. [2]F(1.11)=6.50. p=.027 [3]F(1.4)=39.15. p=.003 [4]F(1.4)=235.73. p=<.001 [5]F(1.4)=1..06. p=.022 [6]F(1.4)=22.60. p=.009 [7]F(1.11)=5.19. p=.044

Table S8: T-test analysing association between mean image quality and education of personnel

Feature	Quality aspect	t-value	Degrees of freedom	p-value	groups	Total quality	std
Education of personnel							
Radiologist followed a course on prostate MRI[1]	Total bp quality	-2.47	11	.031	No	4.77/8	.16
					Yes	5.92/8	.63
	Total T2W quality	-2.72	11	.020	No	2.41/4	.11
					Yes	3.14/4	.37
	T2W axial: adequate SNR	-3.37	11	.006	No	.42/1	.09
					Yes	.76/1	.14
	T2W axial: ability to delineate	-1.67	11	.124	No	.55/1	.02
					Yes	.72/1	.14
	T2W axial: absence of artefacts	-.78	11	.451	No	.79/1	.15
					Yes	.85/1	.09
	T2W sag/cor: ability to delineate	-1.89	11	.086	No	.65/1	.19
					Yes	.81/1	.10
	Total DWI quality	-1.60	11	.139	No	2.36/4	.27
					Yes	2.78/4	.35
	DWI: adequate SNR high b-value	-1.95	11	.077	No	.24/1	.11
					Yes	.50/1	.17
	DWI: adequate range of contrast	-.19	11	.852	No	.72/1	.09
					Yes	.74/1	.16
	DWI: absence of artefacts[2]	-.31	1.06	.804	No	.69/1	.22
					Yes	.74/1	.09
	DWI in plane matching with T2W[3]	-.52	1.02	.695	No	.71/1	.25
					Yes	.80/1	.05
Radiographer followed a course on prostate MRI in addition to the radiologist	Total mp quality[4]	-1.18	3.77	.306	No	6.37/10	.64
					Yes	7.26/10	1.21
	Total bp quality	-1.45	9	.181	No	5.63	.51
					Yes	6.16	.66
	Total T2W quality	-2.04	9	.072	No	2.93	.33
					Yes	3.32	.31
					No	.68	.13

	T2W axial: adequate SNR	-1.89	9	.092	Yes	.82	.11
	T2W axial: ability to delineate	-1.85	9	.097	No	.64	.16
					Yes	.78	.09
	T2W axial: absence of artefacts	-.70	9	.504	No	.83	.10
					Yes	.87	.09
	T2W sag/cor: ability to delineate	-1.31	9	.223	No	.77	.10
					Yes	.85	.10
	Total DWI quality	-.61	9	.556	No	2.70	.34
					Yes	2.84	.37
	DWI: adequate SNR high b-value[5]	-.44	6.25	.672	No	.47	.08
					Yes	.52	.23
	DWI: adequate range of contrast[6]	-.10	6.06	.927	No	.73	.21
					Yes	.74	.12
	DWI: absence of artefacts	-1.10	9	.301	No	.71	.11
					Yes	.77	.08
	DWI in plane matching with T2W	-.50	9	.629	No	.79	.04
					Yes	.81	.06
	Total DCE quality[7]	.66	3.37	.555	No	1.15/2	.08
					Yes	1.01/2	.41
	DCE: assessment of enhancement[8]	.24	3.08	.825	No	.60/1	.02
					Yes	.57/1	.21
	DCE: ability to identify[9]	.91	3.99	.415	No	.55/1	.09
					Yes	.44/1	.22

[1] All mp-hospitals have an educated radiologist. Therefore, no influence of this feature on DCE quality could be investigated. [2.3.4.5.6.7] Levene's test for equality of variances was violated for this analysis. Due to this violated assumption. a t statistic not assuming homogeneity of variances was computed. [2]$F(1.2)=4.95$. $p=.048$ [3]$F(1.11)=38.97$. $p=<.001$ [4]$F(1.4)=39.15$. $p=.003$ [5]$F(1.9)=6.30$. $p=0.033$ [6]$F(1.9)=5.94$. $p=.038$ [7]$F(1.4)=235.73$. $p=<.001$ [8]$F(1.4)=13.06$. $p=0.22$ [9]$F(1.4)=22.60$. $p=0.009$

Table S9: Mann-Whitney U-test for associations with image quality

Feature	Quality aspect	U	Exact sig (2 tailed)	groups	Mean rank
MRI manufacturer	Total DWI T2WI: Sag OR Cor: ability to clearly delineate relevant prostate structures	14	.414	Siemens	7.75
				Philips	5.80
	DWI: DWI in plane matching with T2WI (< 5mm at the posterior prostate)	16	.618	Siemens	6.50
				Philips	7.80
Field strength	DWI: DWI in plane matching with T2WI (< 5mm at the posterior prostate)	15	.444	1.5T	6.14
				3T	8.00
Administration of laxative	DWI: DWI in plane matching with T2WI (< 5mm at the posterior prostate)	0	.167	Never	6.00
				Always. …	12.00
